# Supplementary material for: Rapid evolution of mutation rate and spectrum in response to environmental and population-genetic challenges
Source: Nat Commun. 2022 Aug 13;13:4752. doi: 10.1038/s41467-022-32353-6 (PMC9376063; doi:10.1038/s41467-022-32353-6)
Supplement: Supplementary file 1 — Supplementary Information [file 41467_2022_32353_MOESM1_ESM.pdf]

Supplementary Information

Rapid evolution of mutation rate and spectrum in response to environmental and population-genetic challenges

Wei, Ho et al.

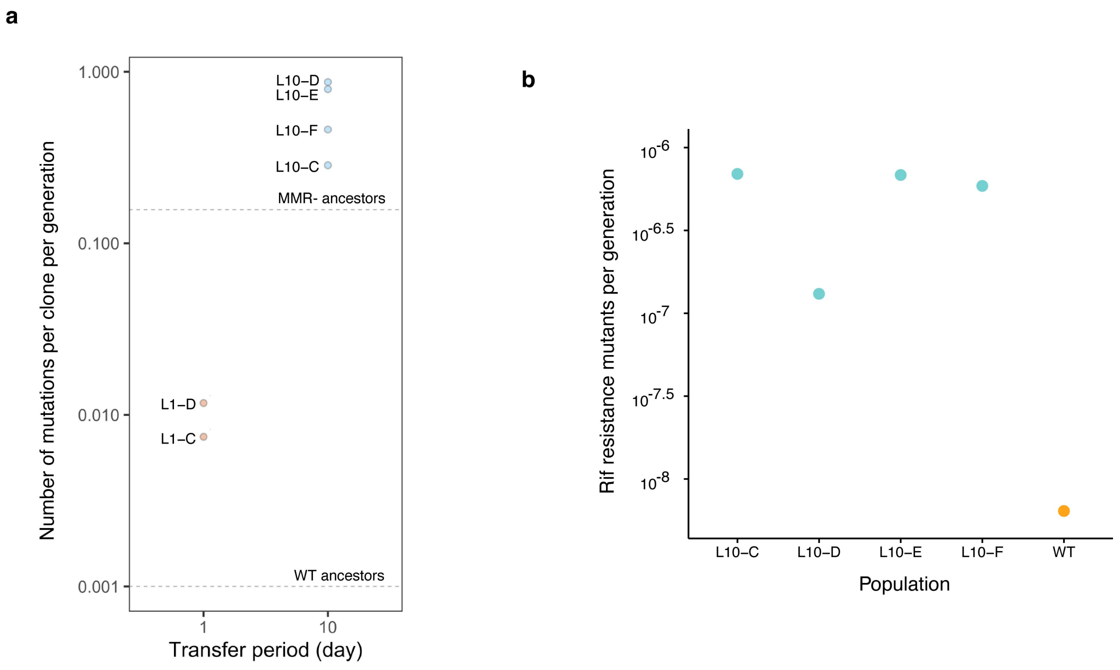

Supplementary Figure 1. **High rate of genomic evolution and mutation rate in L10 WT populations. a** Comparison of the rates of genomic evolution across 1000 days (measured by the number of mutations per clone per generation) in L1 WT populations (C and D) and L10 WT population (C, D, E, and F). Mutation rates (measured by the number of mutations per clone per generation) of WT and MMR- ancestor were also plotted (grey dashed lines). **b** Mean mutation rates conferring rifampicin (rif) resistance estimated by fluctuation tests in clones from L10 WT populations that have evolved for 1000 days and WT (n = 2 per population). Source data are provided as a Source Data file (Data 4).

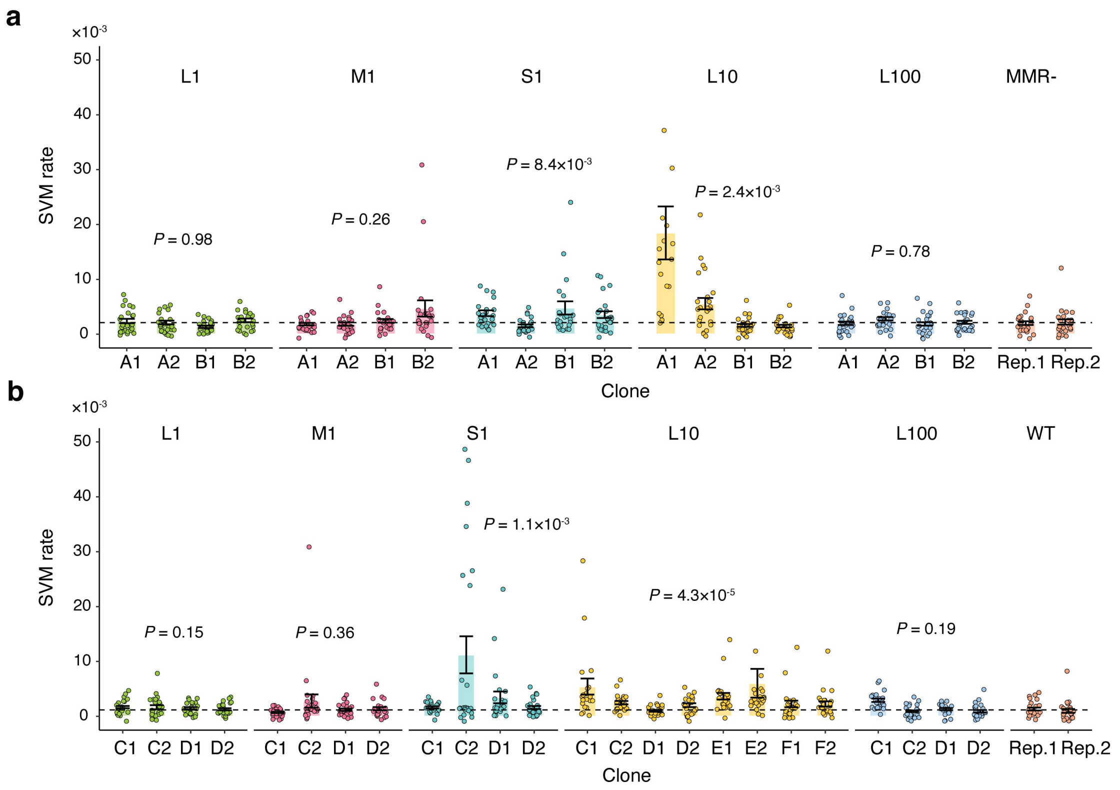

Supplementary Figure 2. **Structural variation mutation (SVM) rates.** Mean SV mutation-rates for (**a**) MMR- and (**b**) WT populations are plotted. *p*-values are based on unpaired, two-sided *t*-tests comparing testing MA lines and the corresponding ancestral MA lines. n = 26, 22, 24, 24, 25, 24, 24, and 25 (L1-A1, A2, B1, B2, C1, C2, D1, and D2, respectively); n = 24, 24, 22, 23, 25, 24, 22, and 24 (M1-A1, A2, B1, B2, C1, C2, D1, and D2, respectively); n = 22, 24, 22, 25, 23, 24, 24, and 24 (S1-A1, A2, B1, B2, C1, C2, D1, and D2, respectively); n = 17, 24, 24, 24, 20, 23, 24, 23, 23, 24, 22, and 24 (L10-A1, A2, B1, B2, C1, C2, D1, D2, E1, E2, F1, and F2, respectively); n = 24, 24, 25, 24, 22, 25, 22, and 23 (L100-A1, A2, B1, B2, C1, C2, D1, and D2, respectively); n = 23 and 24 (WT); n = 24 and 24 (MMR-). Dash lines are average rates of (**a**) MMR- or (**b**) WT ancestral clones. Data are presented as mean values +/- S.E.M.. Source data are provided as a Source Data file (Data 5).

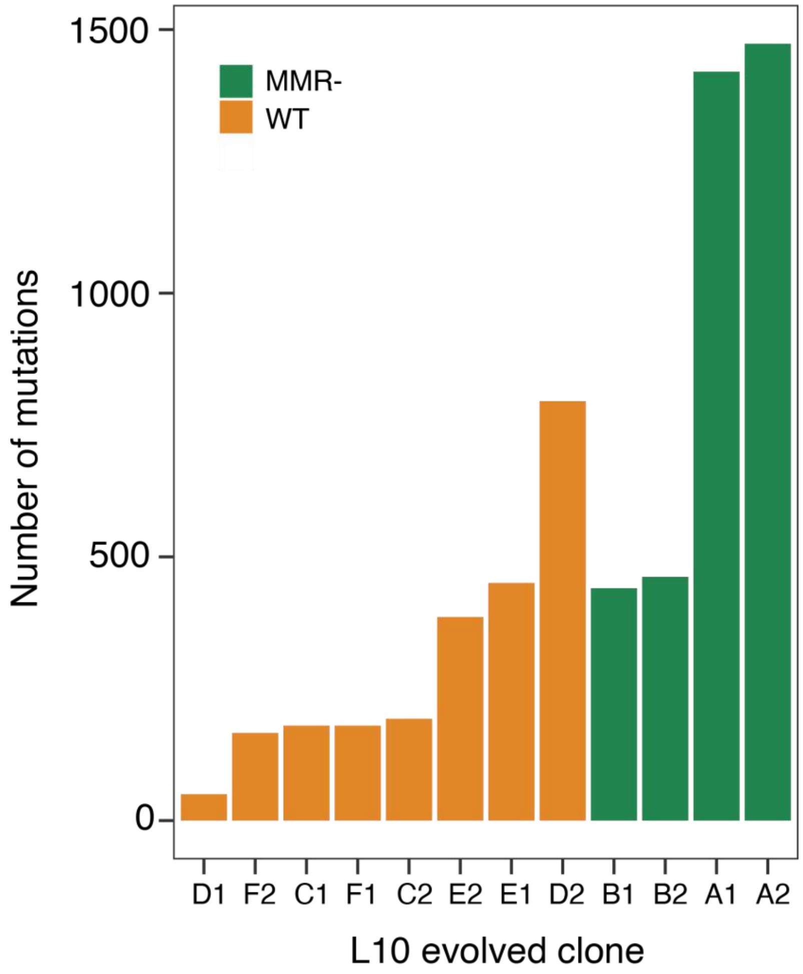

Supplementary Figure 3. **Number of mutations in L10 MA ancestor.** Y-axis shows the number of mutations in the clones isolated from evolved L10 populations with WT (orange) and MMR- background (green). WT clones evolved a larger number of mutations except for D1; the number of mutations in B1, B2, and D2 approached or even exceeded the number of mutations observed in MMR- evolved clones. Source data are provided as a Source Data file (Data 6).

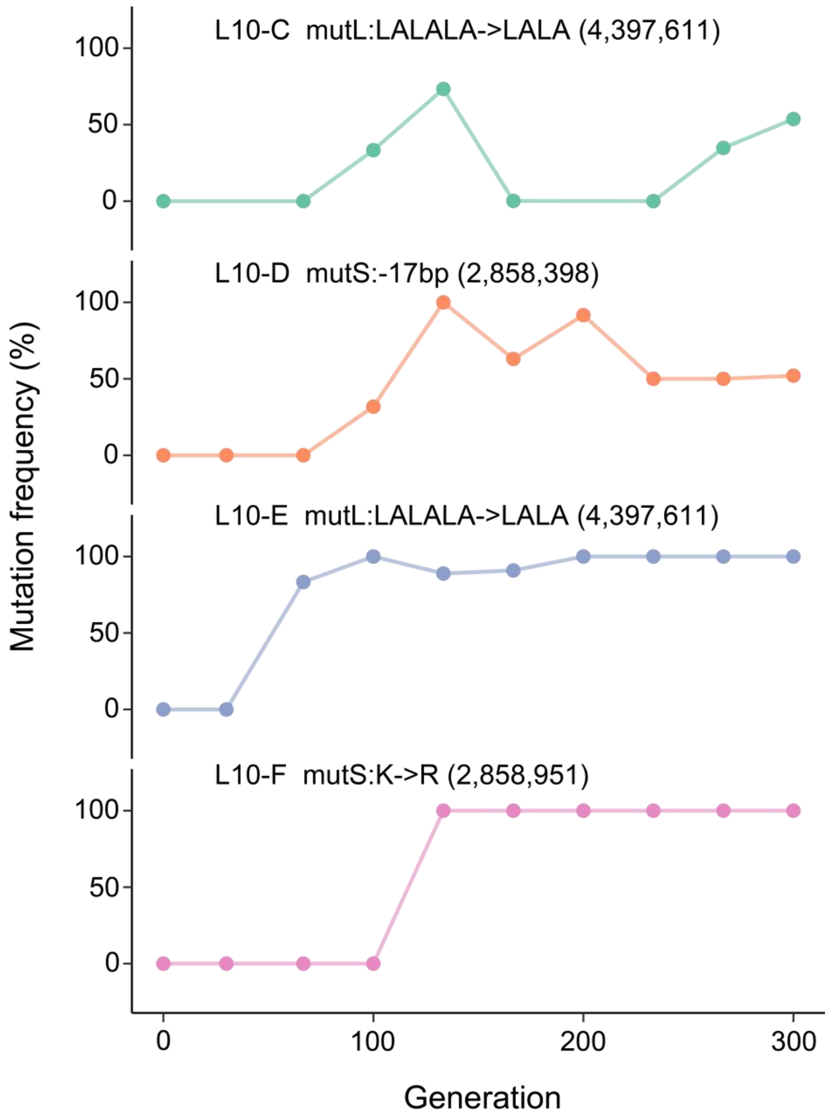

Supplementary Figure 4. **Trajectories of candidate hypermutators estimated from longitudinally-collected whole-population sequencing data.** The feature of mutations and the belonging population are noted in the title. Source data are provided as a Source Data file (Data 7).

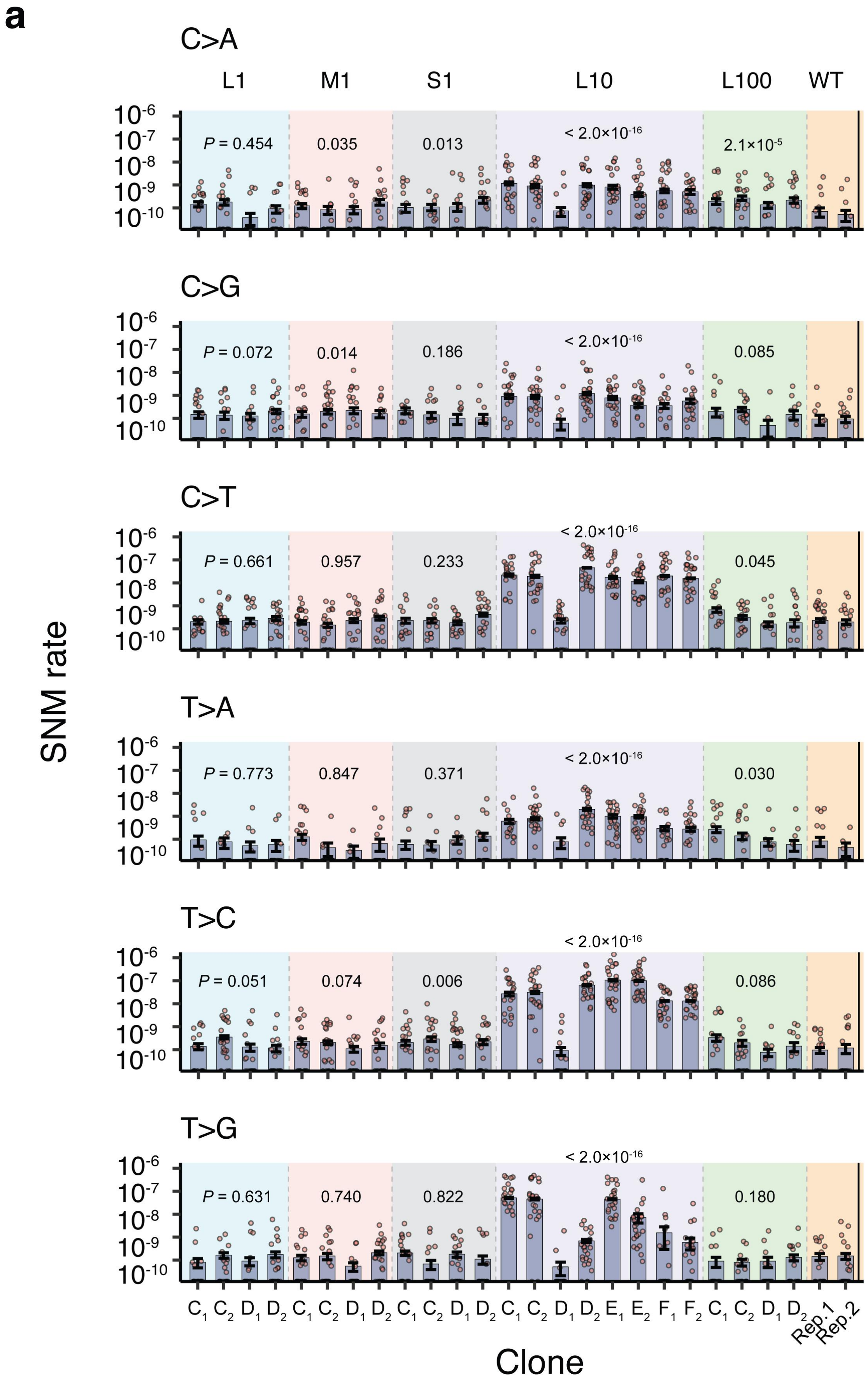
a
C>A
C>G
C>T
T>A
T>C
T>G
L1
M1
S1
L10
L100
WT
SNM rate
P = 0.454
0.035
0.013
< 2.0×10-16
2.1×10-5
P = 0.072
0.014
0.186
0.085
P = 0.661
0.957
0.233
0.045
P = 0.773
0.847
0.371
0.030
P = 0.051
0.074
0.006
0.086
P = 0.631
0.740
0.822
0.180
C1 C2 D1 D2 C1 C2 D1 D2 C1 C2 D1 D2 C1 C2 D1 D2 E1 E2 F1 F2 C1 C2 D1 D2 Rep.1 Rep.2
Clone

b

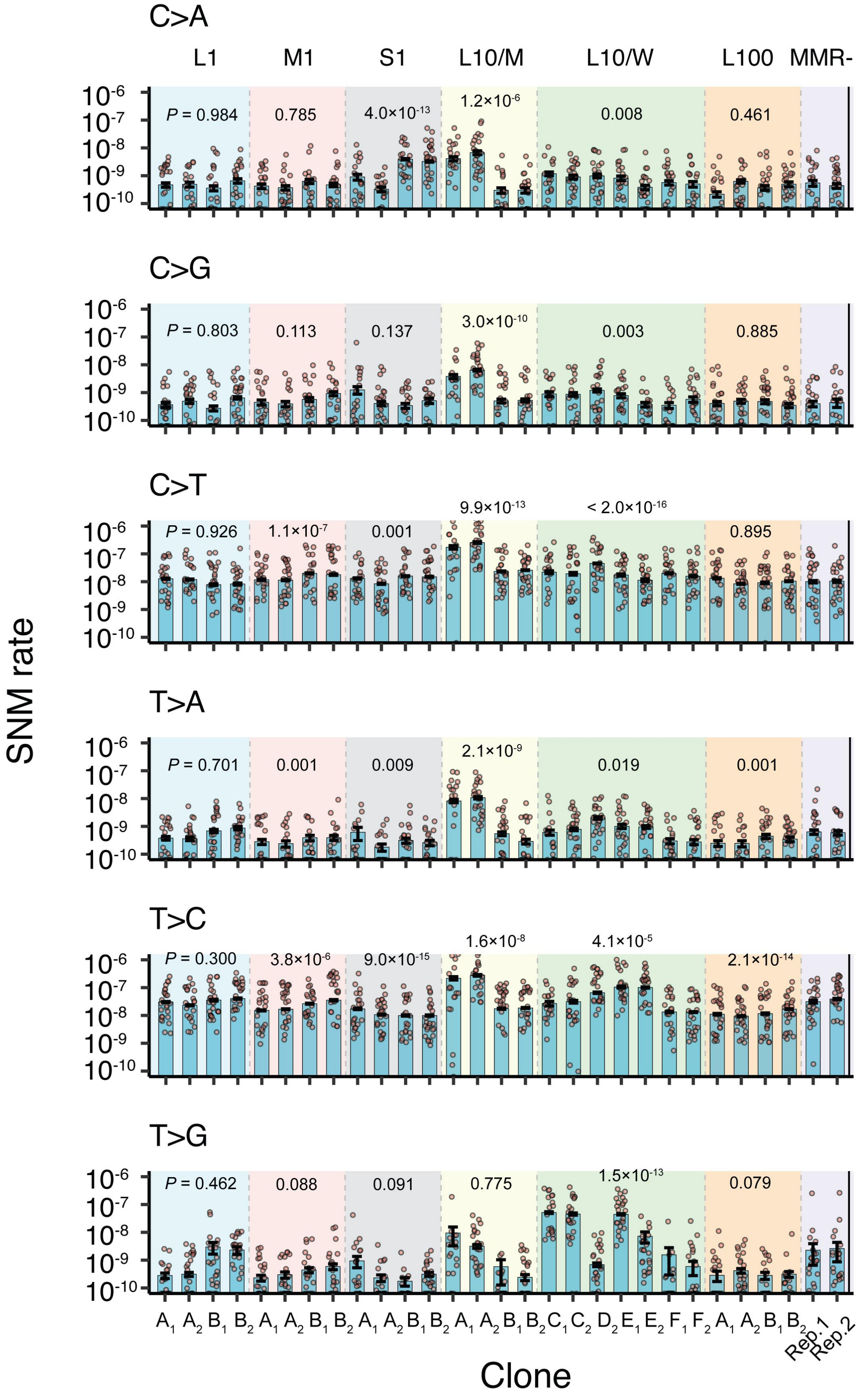
C>A
L1
M1
S1
L10/M
L10/W
L100
MMR-
P = 0.984
0.785
4.0×10-13
1.2×10-6
0.008
0.461
C>G
P = 0.803
0.113
0.137
3.0×10-10
0.003
0.885
C>T
P = 0.926
1.1×10-7
0.001
9.9×10-13
< 2.0×10-16
0.895
T>A
P = 0.701
0.001
0.009
2.1×10-9
0.019
0.001
T>C
P = 0.300
3.8×10-6
9.0×10-15
1.6×10-8
4.1×10-5
2.1×10-14
T>G
P = 0.462
0.088
0.091
0.775
1.5×10-13
0.079
SNM rate
Clone
Rep.1
Rep.2

Supplementary Figure 5. **Mutation rates of six categories of SNM in evolved populations.** Each of six categories of SNM is labeled at the top (C>A, C>G, C>T, T>A, T>C, T>G)**. a** shows the comparison among WT evolved populations and the WT ancestor. **b** shows the comparison among MMR- evolved populations (or the L10 WT populations with identified MMR-related hypermutator alleles) and the MMR- ancestor. L10/M is L10 populations starting from MMR- ancestor, and L10/W is L10 populations with WT background that evolved to have particular high mutation rates. Sample sizes are consistent with Figure 2. Data are presented as mean values +/- S.E.M.. *P*-values were acquired by two-tailed unpaired *t*-tests contrasting MA lines from evolved populations and MA lines from the ancestor. Source data are provided as a Source Data file (Data 8).

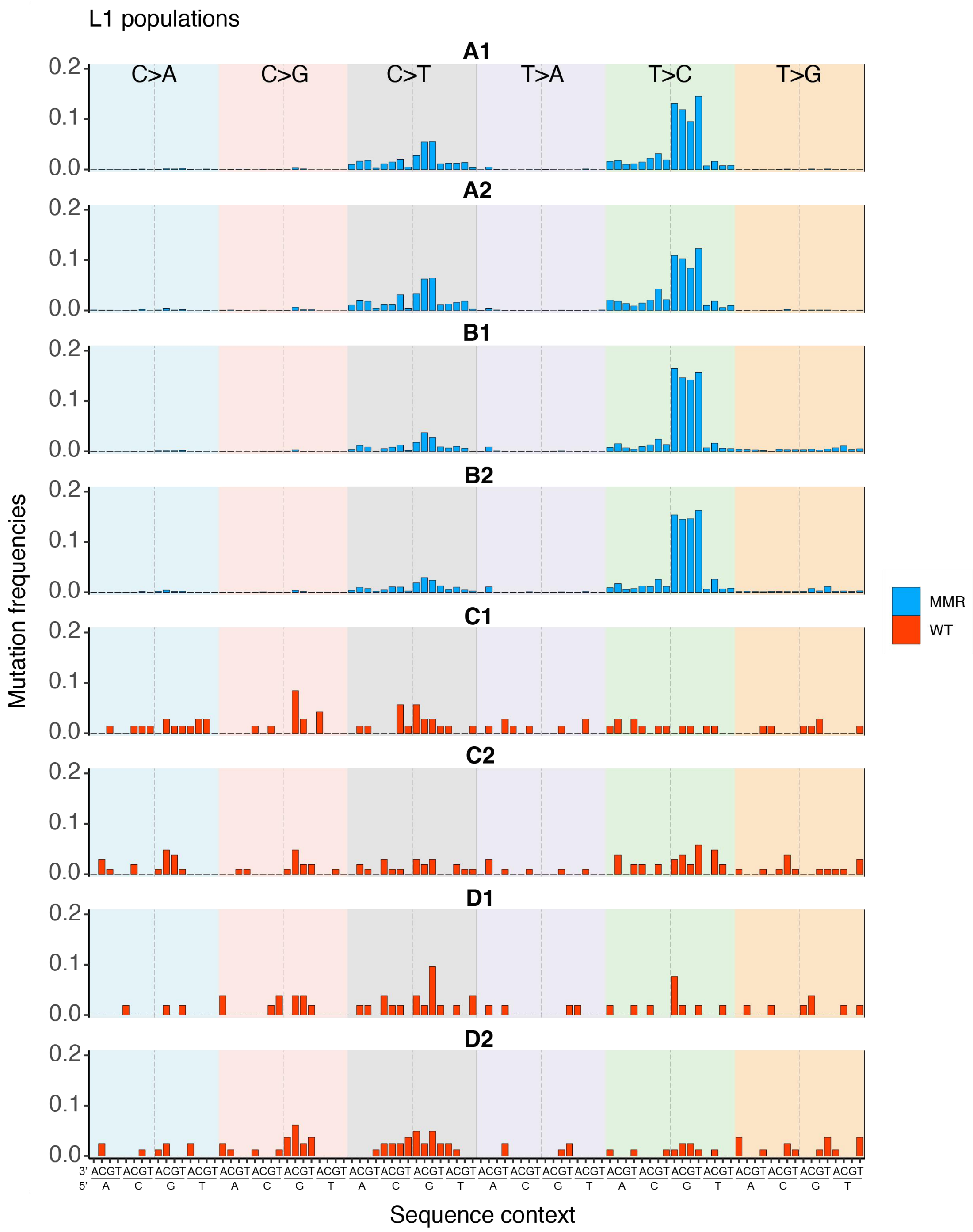
L1 populations
A1
A2
B1
B2
C1
C2
D1
D2
C>A
C>G
C>T
T>A
T>C
T>G
0.2
0.1
0.0
Mutation frequencies
Sequence context
MMR
WT
3'
5'
ACGT
A
C
G
T

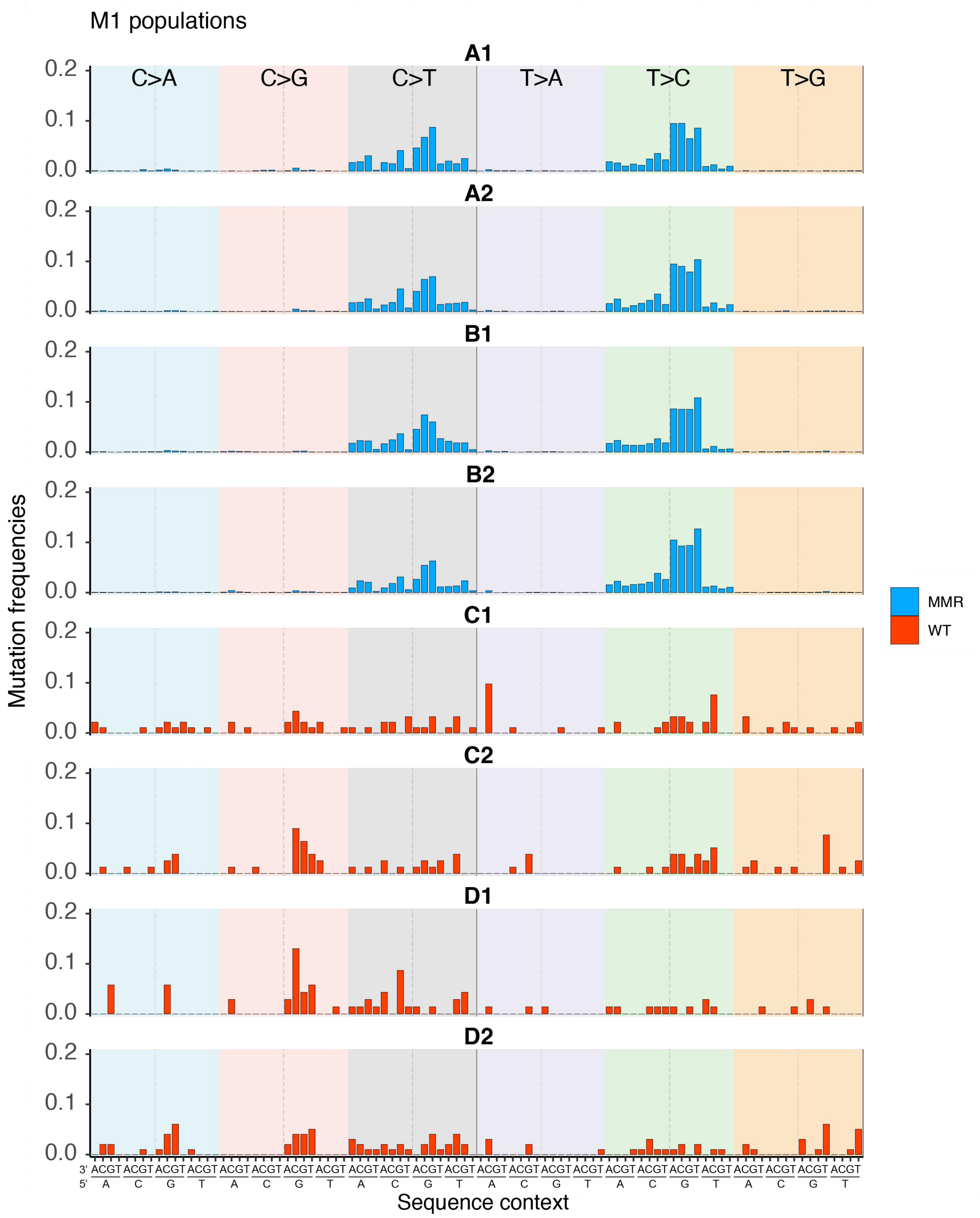

M1 populations
A1
A2
B1
B2
C1
C2
D1
D2
C>A
C>G
C>T
T>A
T>C
T>G
0.2
0.1
0.0
Mutation frequencies
MMR
WT
3'
5'
ACGT
A
C
G
T
Sequence context

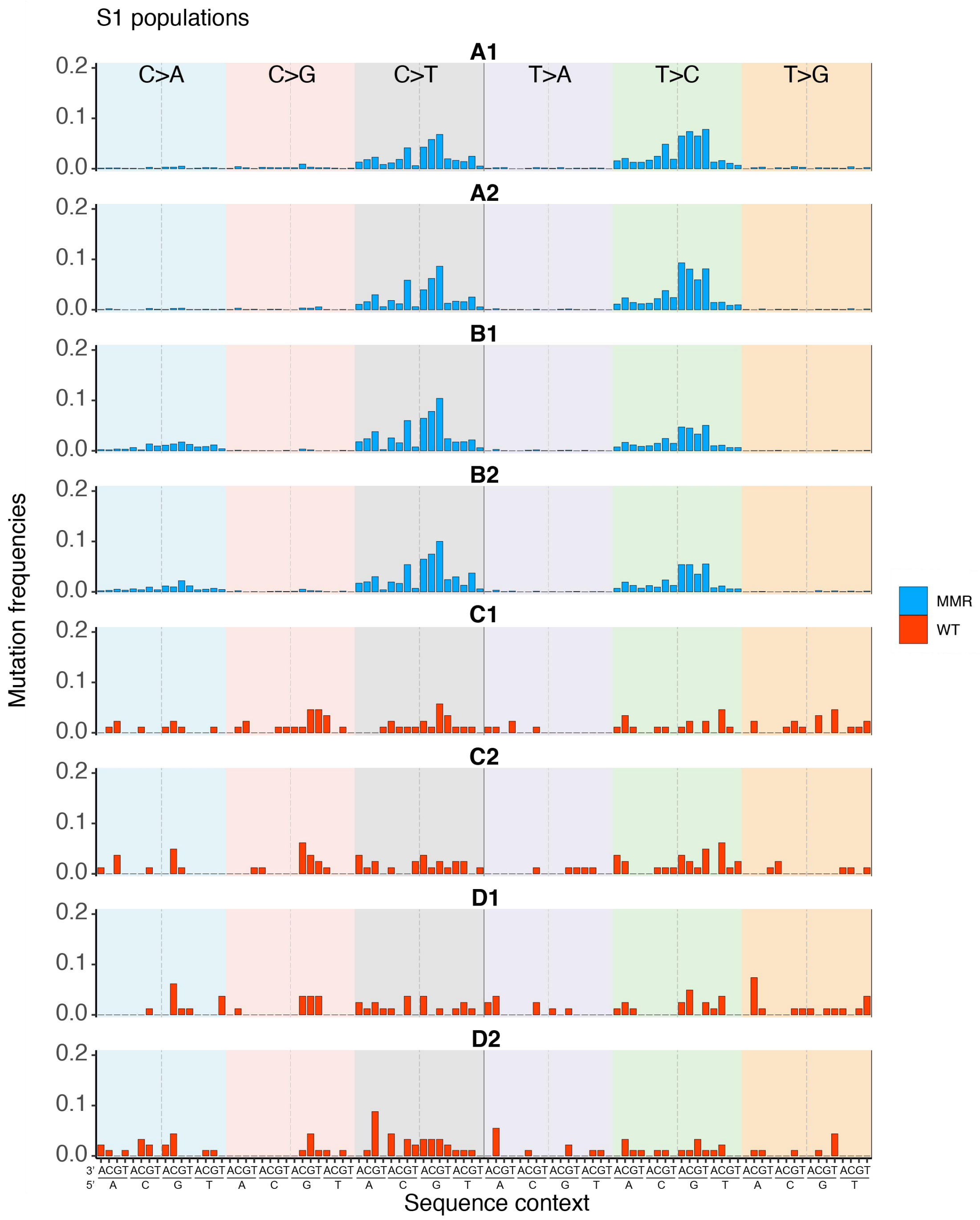
S1 populations
A1
A2
B1
B2
C1
C2
D1
D2
C>A
C>G
C>T
T>A
T>C
T>G
0.2
0.1
0.0
Mutation frequencies
Sequence context
3'
5'
ACGT
A
C
G
T
MMR
WT

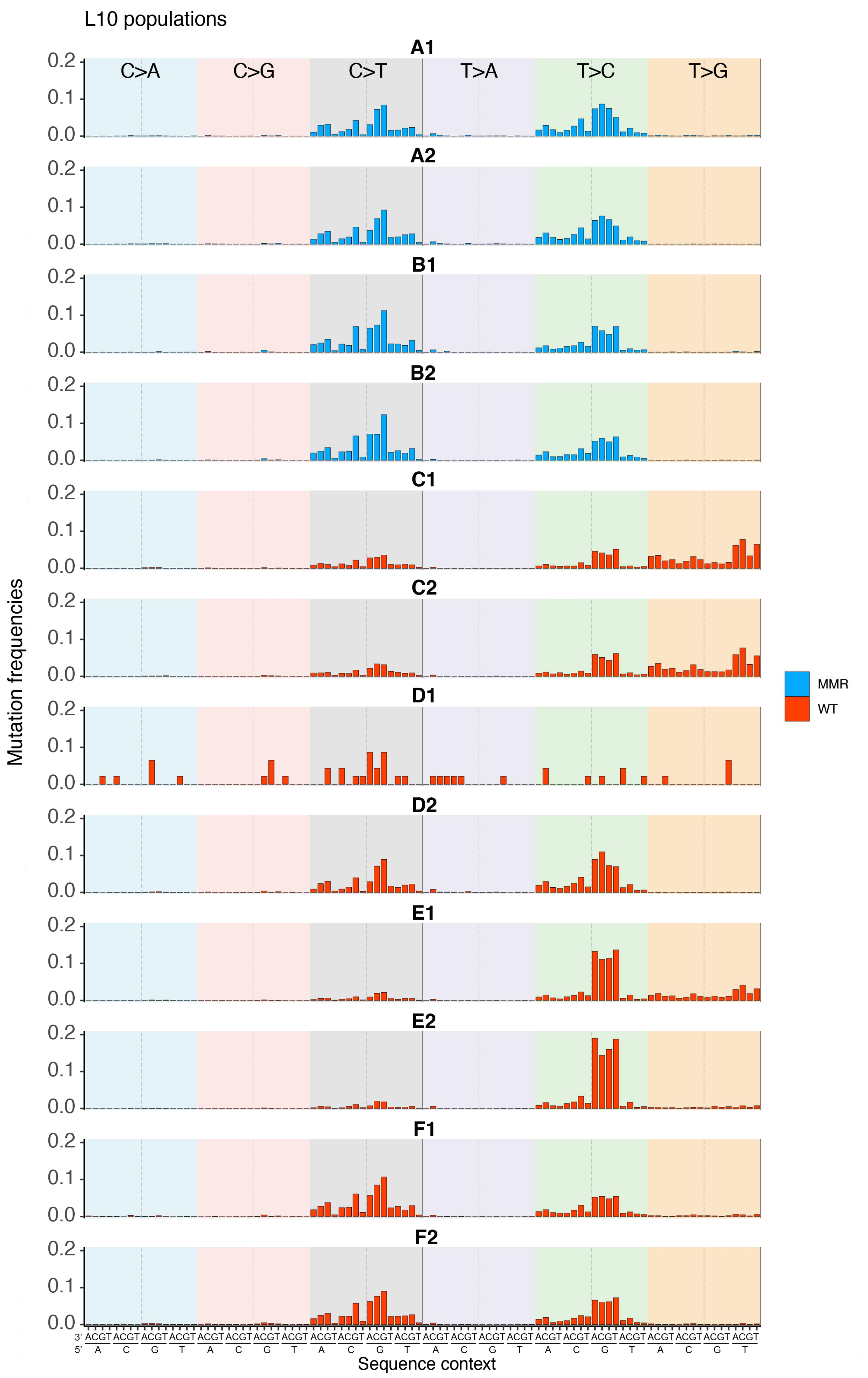

L10 populations
A1
A2
B1
B2
C1
C2
D1
D2
E1
E2
F1
F2
C>A
C>G
C>T
T>A
T>C
T>G
0.2
0.1
0.0
Mutation frequencies
Sequence context
3'
5'
ACGT
A
C
G
T
MMR
WT

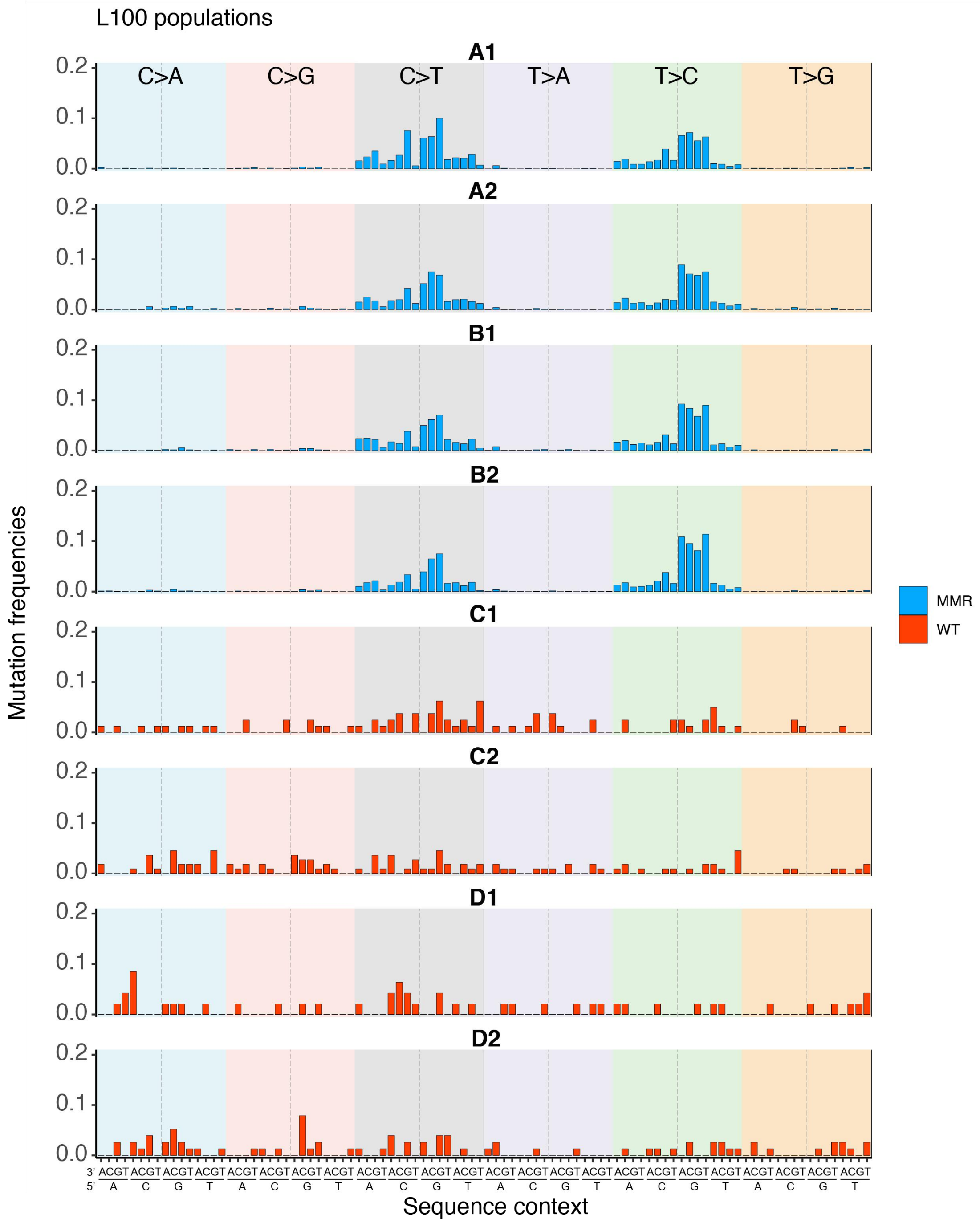
L100 populations
A1
A2
B1
B2
C1
C2
D1
D2
C>A
C>G
C>T
T>A
T>C
T>G
0.2
0.1
0.0
Mutation frequencies
MMR
WT
3'
5'
ACGT
A
C
G
T
Sequence context

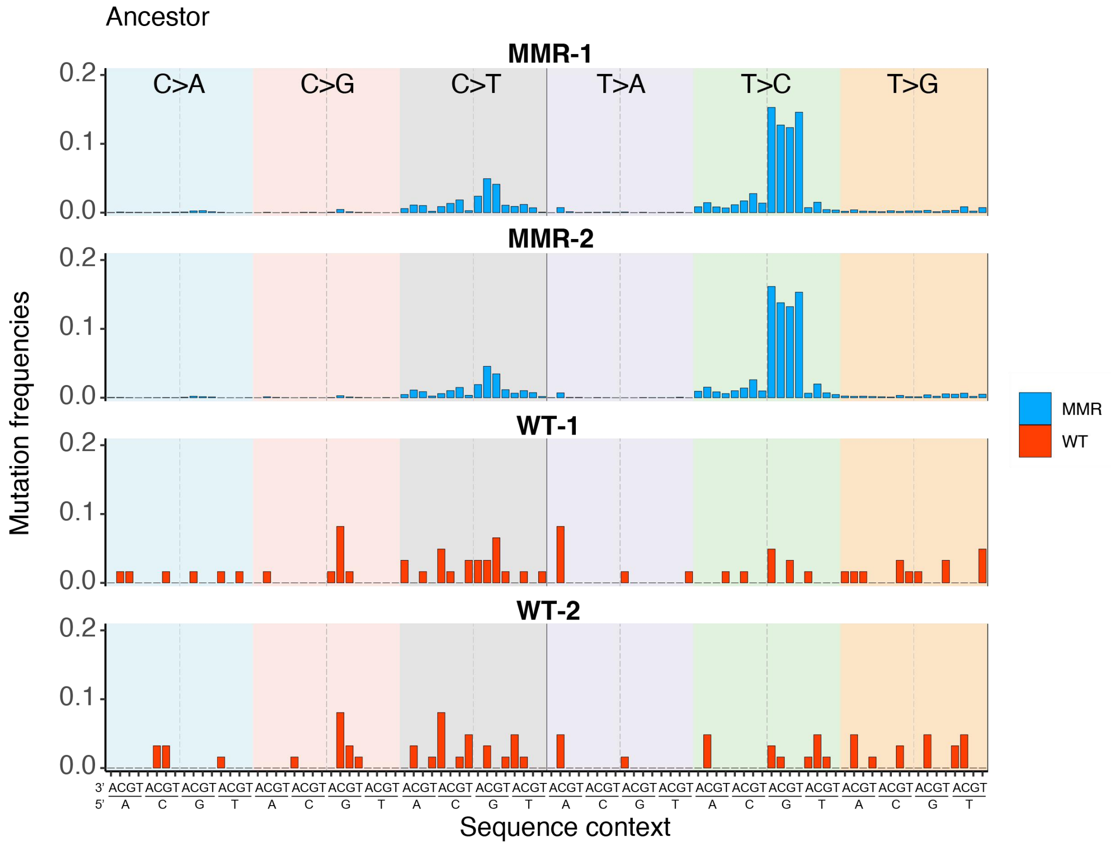

Supplementary Figure 6. **The 96-class contextual mutational spectra of the evolved populations and the ancestors.** Each bar represents a class; each class represents a single nucleotide change (one of six colored blocks; labels on the top) with the context of 3′ and 5′ flanking nucleotide (ticks at the bottom). Source data are provided as a Source Data file (Data 3).

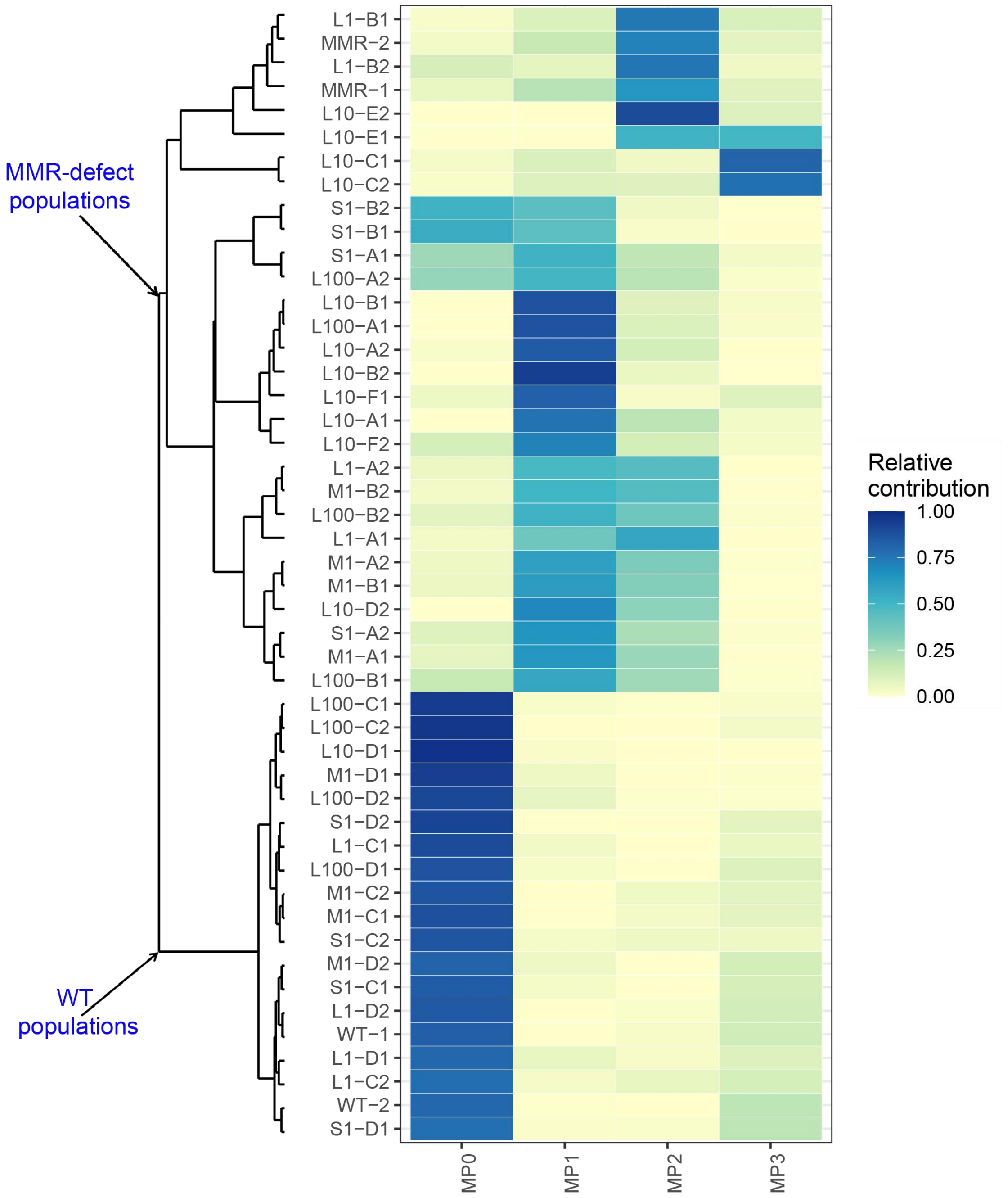

Supplementary Figure 7. **Relative mutation contribution of four mutation patterns (MPs) based on the dimensionality reduction analysis**. Each row represents a clone from a evolved population or an ancestor (the labels same as Fig. 1). The dendrogram on the left summarizes the similarity among the evolved populations and the ancestors. MP0 closely resembles the mutation patterns of WT populations; MP1, MP2, and MP3 are mainly related to evolved populations in the MMR- background and the L10 WT populations with identified MMR-related hypermutator alleles. Source data are provided as a Source Data file (Data 3).

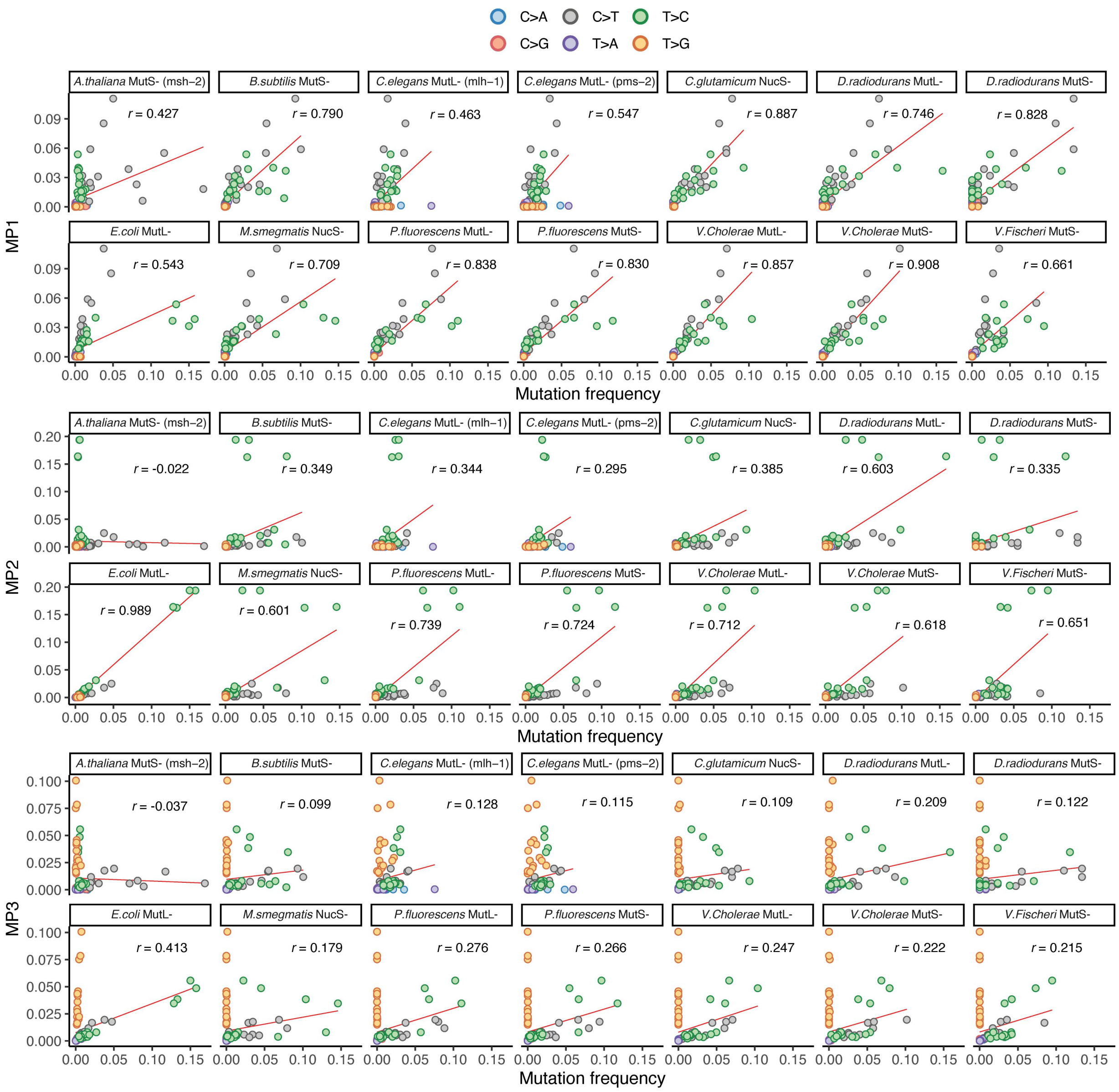

Supplementary Figure 8. **Correlations between the mutation frequencies of six SNM categories in each of the three mutation patterns (MP1, MP2, and MP3) and the mutation frequencies in other MMR-defect other species (noted in the top box).** *r*: Pearson's correlation coefficient. Source data are provided as a Source Data file (Data 3).

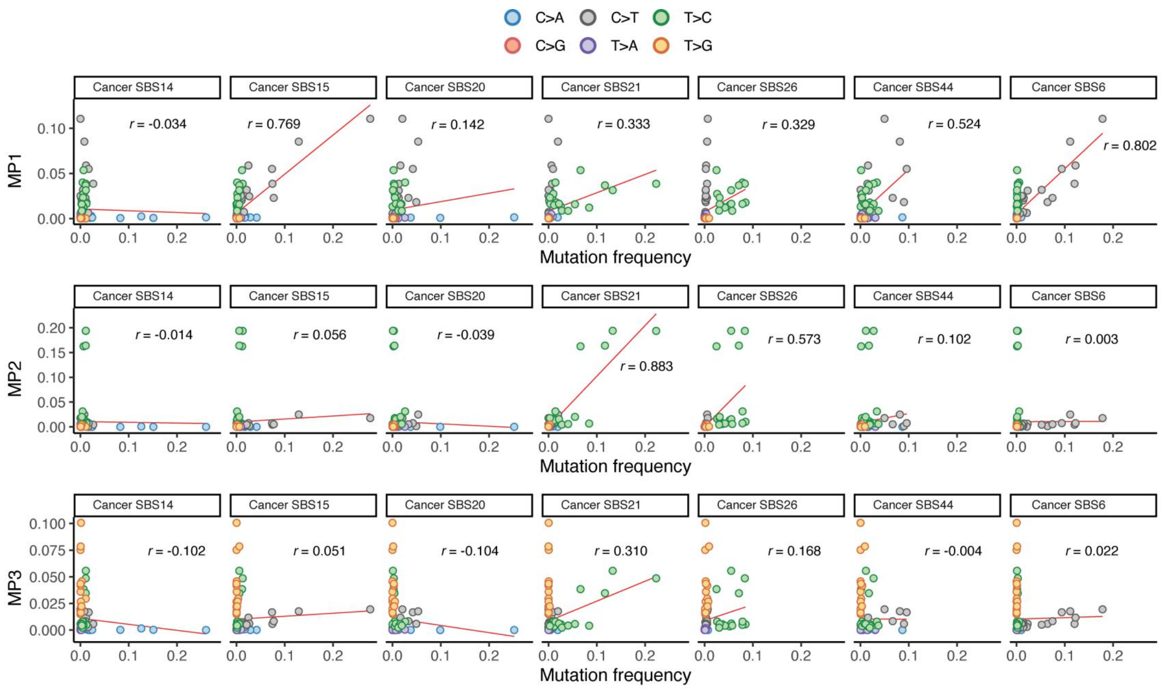

Supplementary Figure 9. **Correlations between the mutation frequencies of six SNM categories in each of the three mutation patterns (MP1, MP2, and MP3) and the mutation frequencies in the single base substitution (SBS) signatures associated with MMR defected cancers in the COSMIC database (noted in the top box).** *r*: Pearson's correlation coefficient. Source data are provided as a Source Data file (Data 3).
