# Supplementary material for: Rapid evolution of mutation rate and spectrum in response to environmental and population-genetic challenges
Source: Nat Commun. 2022 Aug 13;13:4752. doi: 10.1038/s41467-022-32353-6 (PMC9376063; doi:10.1038/s41467-022-32353-6)
Supplement: Supplementary file 3 — Description of Additional Supplementary Files [file 41467_2022_32353_MOESM3_ESM.pdf]

Description of Additional Supplementary Files

File Name: Supplementary Data 1
Description: Summary of MA lines and rates of mutations identified in MA experiments.

File Name: Supplementary Data 2
Description: List of SNM mutations detected in the MA experiments.

File Name: Supplementary Data 3
Description: List of small indels detected in the MA experiments.

File Name: Supplementary Data 4
Description: List of structural variations detected in the MA experiments.

File Name: Supplementary Data 5
Description: Antimutator candidates in MMR- populations, which are on genes related to DNA replication or repair and identified as being fixed within evolved populations or subpopulations.
