# Supplementary material for: Rapid evolution of mutation rate and spectrum in response to environmental and population-genetic challenges
Source: Nat Commun. 2022 Aug 13;13:4752. doi: 10.1038/s41467-022-32353-6 (PMC9376063; doi:10.1038/s41467-022-32353-6)
Supplement: Supplementary file 4 — Supplementary Data 1 [file 41467_2022_32353_MOESM4_ESM.docx]

Supplementary Data 1. **Summary of MA lines and rates of mutations identified in MA experiments.**

| ***Clone*** | ***Description*** | ***Generations per line*** | **No. of MA lines***d* | **SNM rate (×10***10***)***e* | **SIM rate** **(×10***11***)***e* | ***SVM rate (×10^4^)^f^*** |
| --- | --- | --- | --- | --- | --- | --- |
| WT-1 | WT, isolate 1 | 1,730 | 23 | 3.62 | 6.53 | 13.32 |
| WT-2 | WT, isolate 2 | 1,694 | 25 | 3.29 | 6.09 | 9.84 |
| MMR-1 | MMR, isolate 1 | 1,580 | 25 | 224.21 | 498.37 | 20.31 |
| MMR-2 | MMR, isolate 2 | 1,698 | 25 | 265.06 | 509.26 | 22.33 |
|  | MMR- populations |  |  |  |  |  |
| L1-A1 | Large population A, isolate 1 | 1,626 | 26 | 221.73 | 305.30 | 24.13 |
| L1-A2 | Large population A, isolate 2 | 1,657 | 24 | 183.07 | 307.92 | 21.51 |
| L1-B1 | Large population B, isolate 1 | 1,611 | 25 | 236.15 | 521.92 | 13.71 |
| L1-B2 | Large population B, isolate 2 | 1,615 | 25 | 266.10 | 420.54 | 25.29 |
| M1-A1 | Medium population A, isolate 1 | 1,566 | 24 | 142.08 | 268.99 | 18.36 |
| M1-A2 | Medium population A, isolate 2 | 1,464 | 25 | 146.98 | 310.79 | 18.50 |
| M1-B1 | Medium population B, isolate 1 | 1,530 | 23 | 242.35 | 427.04 | 24.36 |
| M1-B2 | Medium population B, isolate 2 | 1,416 | 24 | 277.24 | 536.68 | 46.97 |
| S1-A1 | Small population A, isolate 1 | 1,565 | 22 | 169.82 | 292.95 | 38.33 |
| S1-A2 | Small population A, isolate 2 | 1,662 | 25 | 101.03 | 212.34 | 15.29 |
| S1-B1 | Small population B, isolate 1 | 1,598 | 23 | 153.51 | 265.36 | 48.08 |
| S1-B2 | Small population B, isolate 2 | 1,626 | 25 | 146.7 | 273.26 | 35.67 |
| L10-A1 | Large population A, ten-day cycles, isolate 1 | 557a | 20 | 2074.95 | 621.56 | 184.74 |
| L10-A2 | Large population A, ten-day cycles, isolate 2 | 761b | 24 | 2789.38 | 713.18 | 55.82 |
| L10-B1 | Large population B, ten-day cycles, isolate 1 | 1,477 | 25 | 216.45 | 374.80 | 16.08 |
| L10-B2 | Large population B, ten-day cycles, isolate 2 | 1,581 | 25 | 232.22 | 423.98 | 15.02 |
| L100-A1 | Large population A, 100-day cycles, isolate 1 | 1,469 | 25 | 128.24 | 288.37 | 20.13 |
| L100-A2 | Large population A, 100-day cycles, isolate 2 | 1,577 | 24 | 98.14 | 215.88 | 28.27 |
| L100-B1 | Large population B, 100-day cycles, isolate 1 | 1,487 | 26 | 111.94 | 200.94 | 18.83 |
| L100-B2 | Large population B, 100-day cycles, isolate 2 | 1,567 | 25 | 143.96 | 251.8 | 21.80 |
|  | *WT* populations |  |  |  |  |  |
| L1-C1 | Large population C, isolate 1 | 1,562 | 25 | 4.08 | 12.08 | 16.90 |
| L1-C2 | Large population C, isolate 2 | 1,663 | 25 | 5.64 | 8.68 | 16.54 |
| L1-D1 | Large population D, isolate 1 | 1,463 | 24 | 3.35 | 9.25 | 14.52 |
| L1-D2 | Large population D, isolate 2 | 1,488 | 25 | 4.71 | 9.91 | 12.90 |
| M1-C1 | Medium population C, isolate 1 | 1,656 | 25 | 4.83 | 7.11 | 7.25 |
| M1-C2 | Medium population C, isolate 2 | 1,630 | 25 | 4.14 | 10.09 | 27.86 |
| M1-D1 | Medium population D, isolate 1 | 1,674 | 25 | 3.7 | 10.2 | 13.85 |
| M1-D2 | Medium population D, isolate 2 | 1,658 | 25 | 5.38 | 8.16 | 12.06 |
| S1-C1 | Small population C, isolate 1 | 1,659 | 23 | 5.15 | 14.21 | 16.51 |
| S1-C2 | Small population C, isolate 2 | 1,616 | 25 | 4.53 | 26.21 | 112.13 |
| S1-D1 | Small population D, isolate 1 | 1,663 | 25 | 4.22 | 9.38 | 34.59 |
| S1-D2 | Small population D, isolate 2 | 1,395 | 25 | 6.14 | 29.03 | 16.13 |
|  | *Starvation cycles, WT* |  |  |  |  |  |
| L10-C1 | Large population C, ten-day cycles, isolate 1 | 1,327 | 21 | 510.65 | 513.91 | 54.25 |
| L10-C2 | Large population C, ten-day cycles, isolate 2 | 1,530 | 24 | 489.6 | 476.2 | 25.01 |
| L10-D1 | Large population D, ten-day cycles, isolate 1 | 1,424 | 25 | 2.93 | 6.36 | 9.95 |
| L10-D2 | Large population D, ten-day cycles, isolate 2 | 1,494 | 24 | 580.85 | 372.13 | 20.36 |
| L10-E1 | Large population E, ten-day cycles, isolate 1 | 1,461 | 24 | 838.2 | 942.16 | 36.58 |
| L10-E2 | Large population E, ten-day cycles, isolate 2 | 1,472 | 25 | 597.92 | 758.2 | 60.29 |
| L10-F1 | Large population F, ten-day cycles, isolate 1 | 1,478 | 23 | 180.73 | 422.27 | 22.44 |
| L10-F2 | Large population F, ten-day cycles, isolate 2 | 1,517 | 25 | 154.79 | 409.2 | 22.52 |
| L100-C1 | Large population C, 100-day cycles, isolate 1 | 885c | 23 | 8.9 | 14.45 | 29.29 |
| L100-C2 | Large population C, 100-day cycles, isolate 2 | 1,497 | 25 | 6.36 | 9.83 | 9.35 |
| L100-D1 | Large population D, 100-day cycles, isolate 1 | 1,480 | 24 | 2.99 | 8.9 | 13.21 |
| L100-D2 | Large population D, 100-day cycles, isolate 2 | 1,610 | 24 | 4.43 | 6.42 | 9.18 |

a-c. experiments were ended at days of 30, 31 and 38,respectively.

d. fifteen contaminated lines were removed.

e. rate of mutations per site per generation; lines with odd rates were removed followed by the cutoff of absolute z-score>2.5.

f. rate of mutations per genome per generation; lines with odd rates were removed followed by the cutoff of absolute z-score>2.5.
