# Supplementary material for: Rapid evolution of mutation rate and spectrum in response to environmental and population-genetic challenges
Source: Nat Commun. 2022 Aug 13;13:4752. doi: 10.1038/s41467-022-32353-6 (PMC9376063; doi:10.1038/s41467-022-32353-6)
Supplement: Supplementary file 9 — Reporting Summary [file 41467_2022_32353_MOESM9_ESM.pdf]

nature portfolio

Corresponding author(s): Michael Lynch

Last updated by author(s): Jun 7, 2022

Reporting Summary

Nature Portfolio wishes to improve the reproducibility of the work that we publish. This form provides structure for consistency and transparency in reporting. For further information on Nature Portfolio policies, see our Editorial Policies and the Editorial Policy Checklist.

Statistics

For all statistical analyses, confirm that the following items are present in the figure legend, table legend, main text, or Methods section.

n/a	Confirmed	
☐	☒	The exact sample size (*n*) for each experimental group/condition, given as a discrete number and unit of measurement
☐	☒	A statement on whether measurements were taken from distinct samples or whether the same sample was measured repeatedly
☐	☒	The statistical test(s) used AND whether they are one- or two-sided<br>*Only common tests should be described solely by name; describe more complex techniques in the Methods section.*
☐	☒	A description of all covariates tested
☐	☒	A description of any assumptions or corrections, such as tests of normality and adjustment for multiple comparisons
☐	☒	A full description of the statistical parameters including central tendency (e.g. means) or other basic estimates (e.g. regression coefficient) AND variation (e.g. standard deviation) or associated estimates of uncertainty (e.g. confidence intervals)
☐	☒	For null hypothesis testing, the test statistic (e.g. *F*, *t*, *r*) with confidence intervals, effect sizes, degrees of freedom and *P* value noted<br>*Give P values as exact values whenever suitable.*
☒	☐	For Bayesian analysis, information on the choice of priors and Markov chain Monte Carlo settings
☒	☐	For hierarchical and complex designs, identification of the appropriate level for tests and full reporting of outcomes
☐	☒	Estimates of effect sizes (e.g. Cohen's *d*, Pearson's *r*), indicating how they were calculated

Our web collection on statistics for biologists contains articles on many of the points above.

Software and code

Policy information about availability of computer code

Data collection	No software was used.
Data analysis	We used trimmomatic 0.36 for trimming, BWA 0.7.15 for mapping, and GATK 4.1.8 and Breseq 0.3 for SNP calling. CNVnator 0.3.3, Lumpy 0.2.13, and Breakdancer 1.4.3 were used for structural-mutations calling. We used R package “rSalvador” for estimating rifampicin resistance rates and “MutationalPatterns” for analyzing SNM spectra. Other custom codes are available upon request.

For manuscripts utilizing custom algorithms or software that are central to the research but not yet described in published literature, software must be made available to editors and reviewers. We strongly encourage code deposition in a community repository (e.g. GitHub). See the Nature Portfolio guidelines for submitting code & software for further information.

Data

Policy information about availability of data

All manuscripts must include a data availability statement. This statement should provide the following information, where applicable:

- Accession codes, unique identifiers, or web links for publicly available datasets
- A description of any restrictions on data availability
- For clinical datasets or third party data, please ensure that the statement adheres to our policy

The MA/WGS data generated in this study have been deposited in the NCBI’s short read archive database under PRJNA688002 [https://www.ncbi.nlm.nih.gov/bioproject/?term=PRJNA688002]. The Arabidopsis thaliana genome used in this study are available in the The Arabidopsis Information Resource database [https://www.arabidopsis.org/]. Other genomes are available in the NCBI Genbank under NC_000913.3, NZ_CM000488.1, NC_003450.3, NC_001263.1, NC_001264.1,

NC_018289.1, NC_012660.1, NZ_CP016324.1, NZ_CP016325.1, NC_002505.1, NC_002506.1, NC_006840.2, and NC_006841.2 [https://www.ncbi.nlm.nih.gov/genbank/]. GO-terms are available at gene ontology database [http://geneontology.org/]. SBS patterns of human cancer samples are available in COSMIC database [https://cancer.sanger.ac.uk/cosmic/signatures]. Source data are provided with this paper.

Human research participants

Policy information about studies involving human research participants and Sex and Gender in Research.

Reporting on sex and gender	No human research participants.
Population characteristics	No human research participants.
Recruitment	No human research participants.
Ethics oversight	No human research participants.

Note that full information on the approval of the study protocol must also be provided in the manuscript.

Field-specific reporting

Please select the one below that is the best fit for your research. If you are not sure, read the appropriate sections before making your selection.

☐ Life sciences ☐ Behavioural & social sciences ☒ Ecological, evolutionary & environmental sciences

For a reference copy of the document with all sections, see nature.com/documents/nr-reporting-summary-flat.pdf

Ecological, evolutionary & environmental sciences study design

All studies must disclose on these points even when the disclosure is negative.

Study description	For most combinations of genetic background and transfer schemes, two replicates of evolved populations were studied. For the combination of WT and L10, four evolved populations were studied. Two independently isolated clones were used as MA ancestors for each evolved population, WT ancestor, and MMR- ancestor. For each MA experiment, 25-30 derived lines were used for whole-genome sequencing and mutation-rate estimation.
Research sample	E. coli had been experimentally evolved at 37 °C in LB broth for 1000 days before the clones were isolated as MA ancestors.
Sampling strategy	For each combination of genetic background and transfer scheme, we sampled the two evolved populations with the smallest numbers for the experiment. Because a previous analysis shows that WT, L10 populations have high rates of genomic evolution, we decided to include two additional WT, L10, evolved populations in the study. For each evolved population, we streaked the frozen sample on an agar plate and randomly isolated two colonies as the two independent MA ancestors.
Data collection	DNA sequences were extracted in Lynch Lab, and the high-throughput sequencing was performed in BGI.
Timing and spatial scale	The MA process was performed for 60 days. According to the previous works in the lab, 60 days are needed for the WT ancestor to accumulate sufficient mutations for the statistical analysis.
Data exclusions	The ancestral variants were excluded in mutation-rate calculation. The MA lines with low averaged depths (< 50X) or low coverages (< 30%) were discarded as they were potentially contaminated. The MA lines with a strongly deviated number of mutations (absolute z-scores > 2.5) were also discarded.
Reproducibility	For reproducibility, we worked with at least two evolved populations per a combination of genetic background and transfer scheme. Also, for each evolved population, we performed two MA processes with two independently isolated clones from the population.
Randomization	As we picked the populations with the smallest numbers for the experiments, the sampling process should be random. Colony picking is random as well.
Blinding	We assigned a random letter for each MA experiment. Therefore, the MA experimentalists did not know the exact information on the clones in their experiments.

Did the study involve field work? ☐ Yes ☒ No

Reporting for specific materials, systems and methods

We require information from authors about some types of materials, experimental systems and methods used in many studies. Here, indicate whether each material, system or method listed is relevant to your study. If you are not sure if a list item applies to your research, read the appropriate section before selecting a response.

Materials & experimental systems

n/a	Involved in the study
x	Antibodies
x	Eukaryotic cell lines
x	Palaeontology and archaeology
x	Animals and other organisms
x	Clinical data
x	Dual use research of concern

Methods

n/a	Involved in the study
x	ChIP-seq
x	Flow cytometry
x	MRI-based neuroimaging
